# On the numerical treatment of viscous and convective effects in relative pressure reconstruction methods

**DOI:** 10.1002/cnm.3562

**Published:** 2021-12-17

**Authors:** Douglas R. Q. Pacheco

**Affiliations:** ^1^ Institute of Applied Mathematics Graz University of Technology Graz Austria; ^2^ Present address: Graz Center of Computational Engineering Graz University of Technology Graz Austria

**Keywords:** 4D flow MRI, blood flow, inverse haemodynamics, pressure Poisson equation, pressure reconstruction, relative pressure estimators

## Abstract

The mechanism of many cardiovascular diseases can be understood by studying the pressure distribution in blood vessels. Direct pressure measurements, however, require invasive probing and provide only single‐point data. Alternatively, relative pressure fields can be reconstructed from imaging‐based velocity measurements by considering viscous and inertial forces. Both contributions can be potential troublemakers in pressure reconstruction: the former due to its higher‐order derivatives, and the latter because of the quadratic nonlinearity in the convective acceleration. Viscous and convective terms can be treated in various forms, which, although equivalent for ideal measurements, can perform differently in practice. In fact, multiple versions are often used in literature, with no apparent consensus on the more suitable variants. In this context, the present work investigates the performance of different versions of relative pressure estimators. For viscous effects, in particular, two new modified estimators are presented to circumvent second‐order differentiation without requiring surface integrals. In‐silico and in‐vitro data in the typical regime of cerebrovascular flows are considered, allowing a systematic noise sensitivity study. Convective terms are shown to be the main source of error, even for flows with pronounced viscous component. Moreover, the conservation (often integrated) form of convection exhibits higher noise sensitivity than the standard convective description, in all three families of estimators considered here. For the classical pressure Poisson estimator, the present modified version of the viscous term tends to yield better accuracy than the (recently introduced) integrated form, although this effect is in most cases negligible when compared to convection‐related errors.

## Graphical abstract

This article compares classical and new forms of treating viscous and convective effects in imaging‐based relative pressure reconstruction. Pressure Poisson, Stokes and virtual work‐energy estimators are considered. In‐silico and in‐vitro tests show that integrated variants are in general less accurate, and that the conservation form of convection tends to increase noise sensitivity.

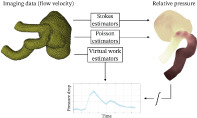

## 1 INTRODUCTION

Blood pressure is not only an important clinical marker, but also a determining factor in the onset and evolution of cardiovascular diseases. This is true not only for the absolute magnitudes of the pressure—for example, measured through catheterization—but also for how the pressure is distributed within a vessel relatively to a reference point in its lumen.^1^ Relative pressure can be understood as a flow's response to inertial effects, viscous resistance and volume forces^2^, ^3^; yet, differently from flow velocities, it cannot be visualized or measured via imaging techniques. Thus, estimating relative arterial pressure from blood‐flow velocities is a relevant task in inverse hemodynamics. As matter of fact, recent studies comparing computed and measured relative pressure show that reconstruction methods can be reliable alternatives to invasive catheter‐based techniques.^4^, ^5^, ^6^, ^7^, ^8^, ^9^ Furthermore, pressure reconstruction is not only relevant in biomedical, but also in general engineering applications using, for example, particle‐based velocimetry.^10^, ^11^, ^12^

Depending on the application, there are essentially two classes of relative pressure estimators: those capable of computing the full pressure field in space and time, and those providing mean pressure differences between selected planes throughout time. Pressure drop markers have a well‐established clinical importance as a metric for stenotic severity,^13^ and can also indicate the risk of growth and rupture in aneurysms.^14^, ^15^, ^16^ Full‐field relative pressure estimators, on the other hand, provide more detailed information with potential diagnostic/prognostic implications: the spatial distribution of intra‐arterial pressure, for instance, is correlated with where an infundibulum is likely to rupture.^7^, ^17^ It is important to remark, however, that a homogeneous pressure rise may also increase the risk of aneurysm rupture but can unfortunately not be captured by relative pressure estimators. This limitation stems from the fact that incompressible flow equations contain only the pressure gradient, so that determining absolute values would require either a reference pressure measurement (at one point at least), or some information on, for example, vessel wall compliance.^4^ Therefore, the pressure estimation methods addressed herein should be understood as relative estimators, an overview of which is found in the recent article by Bertoglio et al.^18^

The most common application of pressure estimators is in determining pressure drops across stenoses, especially in the aorta.^1^, ^2^, ^4^, ^6^, ^19^, ^20^, ^21^ Because of the large flow velocities therein, the pressure gradient tends to be driven predominantly by convective effects.^1^ On the other hand, advances in imaging techniques in the past decade have enabled accurate 4D flow measurements also in smaller vessels,^22^, ^23^, ^24^, ^25^, ^26^, ^27^, ^28^ where viscous effects tend to be more significant. These effects may be even more pronounced in diseased arteries: for instance, typical flow structures in intracranial aneurysms resemble that of lid‐driven cavity flows with strong shear layers.^29^ In fact, relative pressure reconstruction in cerebral vessels has gained increased attention in the last few years,^7^, ^30^, ^31^, ^32^ although further clinical analysis on its impact is still required. Diffusive effects can play a major role not only in low Reynolds regimes, but also in turbulent flows where an eddy viscosity is introduced to model turbulent dissipation,^33^ and pressure estimators incorporating turbulent effects have been recently presented.^21^, ^33^, ^34^

Despite the mathematical simplicity of the viscous term in the (Newtonian) Navier–Stokes system, pressure estimation methods often neglect this term because of its higher‐order velocity derivatives. In the popular pressure Poisson estimator (PPE), for example, second‐order spatial derivatives are present even when using weak formulations.^18^ Therefore, retaining viscous effects normally requires higher‐order interpolation of the velocity data.^2^ Besides, second‐order differentiation can lead to large errors for noisy velocity measurements^33^ and should thus be avoided when possible. Pacheco and Steinbach^35^ modified the PPE to include the viscous contribution using only first‐order derivatives, but the required boundary integral could reduce accuracy in the presence of near‐wall data distortions and imaging artifacts.^6^ The Stokes estimator (STE) recently introduced by Švihlová et al.^36^ and revisited by Bertoglio et al.^18^ can account for viscous effects without second‐order derivatives or boundary operators, but at the cost of quadrupling the number of unknowns in comparison to the PPE. In spite of its higher cost, the Stokes estimator is among the simplest and most accurate techniques for reconstructing relative pressure fields from velocities.

While viscous forces can be troublemakers due to higher‐order differentiation, convective forces can also lead to increased noise sensitivity due to data squaring. The standard form of the convective term contains the product between the velocity field and its spatial gradient, which is why Bertoglio et al.^18^ proposed a modification of the Stokes estimator integrating this term by parts to get rid of velocity derivatives. Also proposed therein is the integral momentum relative pressure (IMRP) estimator where integration by parts is applied to reduce the order of differentiation of both viscous and convective terms. Yet, shortly after that, Marlevi et al.^6^ reported that the surface operator produced by integrating the convective term by parts can deteriorate the method's accuracy, and thus proposed the virtual work‐energy relative pressure (vWERP) estimator keeping the convective term in its non‐integrated form. To the best of the author's knowledge, however, no actual quantitative comparison between IMRP and vWERP estimators is yet available. Moreover, applications of both integrated^18^ and non‐integrated^9^ versions of the Stokes estimator can be found in the recent literature, and only a limited comparison has been reported to date.^18^

Given the importance of both viscous and convective terms for the accuracy of relative pressure estimators, as well as the shortage of comparative studies assessing different ways of treating such terms, this work presents a systematic investigation considering three popular families of estimators in different variants. Classical and state‐of‐the‐art techniques are considered, as well as new modified treatments of the viscous term. For the PPE, a numerical technique is introduced to reconstruct viscous forces without resorting to boundary integrals or second‐order derivatives; for the vWERP estimator, a simple modification based on the rotational form of the momentum equation is proposed to eliminate the viscous boundary term. Different test cases are carried out with increasing complexity, starting from two‐dimensional flow problems with synthetic measurements and then moving to more practical tests considering 4D magnetic resonance imaging (MRI) data. It is important to remark that when using other imaging modalities, such as ultrasound, a three‐dimensional velocity field might not be immediately available but can be reconstructed using appropriate techniques.^37^, ^38^ In that case, the pressure can be estimated either after reconstructing the velocity field, using for example the methods discussed herein, or reconstructed with the velocity. The reader is referred to the recent work of Galarce et al.^38^ for details on state‐of‐the‐art reconstruction techniques from Doppler ultrasound imaging.

## 2 PRELIMINARIES

An assumption common to most pressure estimators is that the flow velocity u and the pressure p satisfy the Navier–Stokes momentum equation:

(1) $$\nabla p=\nabla \cdot S-\rho \frac{\partial u\;}{\partial t}-\left(\rho \nabla u\;\right)u≕\;f\;\left(u\;\right),$$

where ρ is the fluid's density and S is the viscous stress tensor, which under the Newtonian assumption is given by

(2) $$S=2\mu \nabla^{s}u=\mu \left[\nabla u+{\left(\nabla u\;\right)}^{Τ}\right],$$

with μ denoting the dynamic viscosity. The pressure gradient is thus driven by three contributions: viscous, transient and convective forces. Both the viscous and the convective terms have the potential to increase noise sensitivity in pressure reconstruction: the former due to its second‐order derivatives, and the latter because of the “squaring” of the velocity data. The focus of the present work is thus placed on different treatments of these terms.

The conservation of mass for an incompressible flow reads simply

(3) ∇⋅u=0,

which allows different simplifications of the stress divergence ∇⋅S, as well as consistent modifications of the convective term. For the viscous term, the most popular version is the Laplacian form,

(4) $$\nabla \cdot S\;=\mu \nabla \cdot \left[\nabla u+{\left(\nabla u\;\right)}^{Τ}\right]\equiv \mu \left[\nabla^{2}\;u+\nabla \left(\nabla \cdot u\;\right)\right]=\mu \nabla^{2}\;u,$$

with $\nabla^{2}$ denoting the (vector) Laplace operator. Although measured hemodynamic velocities are in practice not divergence‐free—because of measurement and interpolation errors—, the simplification above is still consistent. Also noting that

$$\nabla^{2}u\equiv \nabla \left(\nabla \cdot u\;\right)-\nabla \times \left(\nabla \times u\;\right),$$

where ∇× denotes the curl operator, leads to the rotational variant:

(5) $$-\nabla p=\mu \nabla \times \left(\nabla \times u\;\right)+\rho \frac{\partial \;u\;}{\partial t}+\left(\rho \nabla u\;\right)\;u.$$

Although not so popular in flow simulations due to its somewhat unusual boundary conditions,^39^ this formulation is especially interesting for pressure reconstruction, as will be shown later on.

As for convection, the standard (often called “convective”) form $\left(\rho \nabla u\;\right)\;u\;$ can be recast into the so‐called “divergence form” or “conservation form”:

(6) $$\nabla \cdot \left(\rho u⊗u\;\right),$$

since

$$\rho \nabla \cdot \left(\;u⊗u\;\right)\equiv \left(\rho \nabla u\;\right)\;u+\left(\rho \nabla \cdot u\;\right)\;u.$$

This variant arises, for example, when applying integration by parts to Stokes^18^ or virtual‐work‐based^18^, ^38^ estimators. Although all of these forms are equivalent when considering perfect (divergence‐free) measurements, they can perform very differently in the presence of noisy data.

The focus herein are techniques relying on Lagrangian interpolation of the velocity data, in particular using continuous, piecewise (tri‐)linear finite element spaces $X_{h}$. The interpolated velocity field at time $t=t_{n}$ will be denoted by $\;u^{n}$. A backward finite difference is used for the temporal discretization:

(7) $${\left.\frac{\partial \;u\;}{\partial t}\right|}_{t=t_{n}}\approx \frac{1}{\Delta t}\left(\;u^{n}-u^{n-1}\right),$$

where $\Delta t=t_{n}-t_{n-1}$. Consider a spatial domain $\Omega \in R^{d}$, d=2 or 3, with a boundary Γ≔∂Ω split into three non‐overlapping regions: $\Gamma =\Gamma_{i}\cup \Gamma_{o}\cup \Gamma_{w}$, denoting inlet, outlet and vessel wall, respectively. In stenotic vessels, for instance, an important clinical measure is the mean pressure drop $\mathrm{\delta p}\;\left(t\right)$ across the obstruction:

(8) $$\mathrm{\delta p}={\bar{p}}_{i}-{\bar{p}}_{o}=\frac{1}{|\Gamma_{i}|}\int_{\Gamma_{i}}p\;\mathrm{d\Gamma}-\frac{1}{|\Gamma_{o}|}\int_{\Gamma_{o}}p\;\mathrm{d\Gamma}.$$

When studying other conditions such as aneurysms and infundibula, there is also interest in estimating the actual relative pressure field.^40^ In that case, the vector‐valued momentum Equation (1) becomes an overdetermined system, since the velocity field is already known and one is left with d equations to find a single scalar field. Different estimators arise depending on the method used for solving this inverse problem, as will be seen throughout this work.

## 3 CLASSICAL AND STATE‐OF‐THE ART ESTIMATORS

### 3.1 Pressure Poisson estimators (PPE)

The most classical technique able to fully quantify the pressure field is the so‐called pressure Poisson estimator (PPE). It results from the optimization problem to find the minimum of

$$\frac{1}{2}\int_{\Omega }{\left|\nabla p-\;f\;\left(\;u\;\right)\right|}^{2}\;\mathrm{d\Omega},$$

as shown by Song et al.^41^ Using a variational principle leads to the weak Poisson problem of finding $p\in X_{h}$ such that

(9) $$\int_{\Omega }\nabla q\cdot \nabla p\;\mathrm{d\Omega}=\int_{\Omega }\nabla q\cdot \left(\nabla \cdot S\right)\;\mathrm{d\Omega}-\int_{\Omega }\nabla q\cdot \rho \frac{\partial \;u\;}{\partial t}\;\mathrm{d\Omega}-\int_{\Omega }\nabla q\cdot \left[\left(\rho \nabla u\;\right)\;u\;\right]\mathrm{d\Omega}\;\text{for}\;\mathrm{all}\;q\in X_{h},$$

with consistent Neumann boundary conditions already implied.^35^ Since this is a pure Neumann problem, the pressure at each time is defined up to an arbitrary additive constant. An additional constraint is thus required, for instance enforcing that

(10) $$\int_{\Gamma_{o}}p\;\mathrm{d\Gamma}=0\;\text{for}\;\mathrm{all}\;t.$$

A critical limitation of the standard PPE is the viscous term ∇⋅S containing second‐order spatial derivatives of u. Standard Lagrangian finite elements are only $C^{0}$‐continuous and therefore unable to account for higher‐order derivatives (regardless of the polynomial degree used in the interpolation!). Therefore, computing the viscous term requires either $C^{1}$‐continuous interpolation of the flow data^2^ or finite differences.^33^ To avoid large errors induced by second‐order differentiation, it is also common to simply neglect the viscous term,^18^ due to the typically small viscous contribution in stenotic flows.^1^

To allow capturing viscous effects without computing second‐order derivatives and within a Lagrangian finite element framework, a least‐squares‐like reconstruction step is proposed here for the stress divergence. By projecting the viscous tensor S onto a continuous finite element space before introducing it into the PPE, the resulting tensor $S\in {\left[X_{h}\right]}^{d\times d}$ will lead to a stress divergence ∇⋅S which is piecewise constant, hence non‐vanishing. More precisely, first compute each component $S_{\mathrm{ij}}$, with i,j=1,…,d, such that

(11) $$\int_{\Omega }S_{\mathrm{ij}}\zeta \;\mathrm{d\Omega}=\int_{\Omega }\left(\frac{\partial u_{i}}{\partial x_{j}}+\frac{\partial u_{j}}{\partial x_{i}}\right)\mu \zeta \;\mathrm{d\Omega}\;\text{for}\;\text{all}\;\zeta \in X_{h},$$

with $u_{i}$ denoting the i‐th spatial component of u, and considering the quantities evaluated at the desired time instant $t_{n}$. Then, find $p^{n}\in X_{h}$ such that

(12) $$\begin{array}{l} \int_{\Omega }\nabla q\cdot \nabla p^{n}\;\mathrm{d\Omega}=\int_{\Omega }\nabla q\cdot \left(\nabla \cdot S^{n}\right)\;\mathrm{d\Omega}-\int_{\Omega }\nabla q\cdot \left[\frac{\rho }{\Delta t}\left(u^{n}-u^{n-1}\right)+\left(\rho \nabla u^{n}\right)\;u^{n}\right]\mathrm{d\Omega}\;\text{for}\;\mathrm{all}\;q\in X_{h}. \end{array}$$

Notice that, due to the symmetry of the stress tensor, only $d\left(d+1\right)/2$ (rather than $d^{2}$) scalar projections are needed. In three dimensions, this corresponds to six scalar mass‐matrix systems—all with the same coefficient matrix—that must be solved at each time step. The added computational cost can be greatly mitigated by storing and re‐using the LDL decomposition of the mass matrix. This projected‐stress estimator (PPE${}_{S}$) has the advantage of allowing non‐homogeneous viscosity, as arising from turbulent^33^ or non‐Newtonian^35^ hemodynamic models.

A technique sometimes employed to circumvent second‐order differentiation is integrating the viscous term by parts. This is however not immediately possible in pressure Poisson estimators, since the test functions q already have first‐order derivatives in the weak form. Nonetheless, using the rotational description of the viscous forces (5) actually enables such a step. Notice that

$$-\int_{\Omega }\nabla q\cdot \left[(\nabla \times \left(\nabla \times u\;\right)\right]\mathrm{d\Omega}=-\int_{\Omega }\left(\nabla \times \nabla q\right)\cdot \left(\nabla \times u\;\right)\mathrm{d\Omega}+\int_{\Gamma }\left(\;n\times \nabla q\right)\cdot \left(\nabla \times u\;\right)\mathrm{d\Gamma}=\int_{\Gamma }\left(\;n\times \nabla q\right)\cdot \left(\nabla \times u\;\right)\mathrm{d\Gamma},$$

since ∇×∇q≡0 in $\;L^{2}\left(\Omega \right)$ even for standard $H^{1}\left(\Omega \right)$ functions.^42^ This leads to the boundary vorticity or integrated pressure Poisson estimator (PPE${}_{\omega }$) recently proposed by Pacheco and Steinbach,^35^ seeking $p^{n}\in X_{h}$ such that

(13) $$\int_{\Omega }\nabla q\cdot \nabla p^{n}\;\mathrm{d\Omega}=\mu \int_{\Gamma }\left(\;n\times \nabla q\right)\cdot \left(\nabla \times u^{n}\right)\;\mathrm{d\Gamma}-\int_{\Omega }\nabla q\cdot \left[\frac{\rho }{\Delta t}\left(\;u^{n}-u^{n-1}\right)+\left(\rho \nabla u^{n}\right)\;u^{n}\right]\mathrm{d\Omega}\;\text{for}\;\mathrm{all}\;q\in X_{h},$$

where n denotes the unit outward normal vector on ∂Ω. Although simple and computationally cheap, this estimator relies on a surface integral, a type of operator which is sometimes avoided in MRI‐based pressure reconstruction due to typically inaccurate near‐wall flow measurements.^6^ The performance of these two variants (PPE${}_{S}$ and PPE${}_{\omega }$) will be compared in Section 4, including a third variant PPE${}_{\mathrm{div}}$ using the divergence form (6) of the convective term.

### 3.2 Stokes estimators (STE)

The overdeterminancy of (1) can be handled by introducing a vector‐valued unknown and an additional scalar equation. In that context, and to circumvent the higher regularity requirements of the classical PPE, Švihlová et al.^36^ proposed perturbing the momentum equation with the Laplacian of an (artificial) incompressible velocity field w. This leads to the Stokes problem of finding $\left(\;w,p\right)$ such that

(14) $$-\nabla^{2}\;w+\nabla p=\nabla \cdot S-\rho \frac{\partial \;u\;}{\partial t}-\left(\rho \nabla u\;\right)\;u\;\text{in}\;\Omega ,$$

(15) ∇⋅w=0inΩ,

(16) w=0onΓ.

When using finite element methods to solve this problem, one can take advantage of the variational framework and use integration by parts to get rid of the second‐order derivatives in ∇⋅S. In fact, it is also possible to eliminate the derivative in the convective term: the integrated Stokes estimator (STE${}_{\mathrm{int}}$) proposed by Bertoglio et al.^18^ seeks $\left(\;w^{n},p^{n}\right)\in {\left[X_{h}\right]}^{d}\times X_{h}$, with ${\left.\;w^{n}\right|}_{\Gamma }=\;0\;$, such that

(17) $$\int_{\Omega }\nabla v:\nabla w^{n}\;\mathrm{d\Omega}-\int_{\Omega }p^{n}\nabla \cdot v\;\mathrm{d\Omega}=\int_{\Omega }\nabla v:\left(\rho \;u^{n}⊗u^{n}-\mu \nabla u^{n}\right)\mathrm{d\Omega}-\frac{\rho }{\Delta t}\int_{\Omega }\;v\cdot \left(\;u^{n}-u^{n-1}\right)\;\mathrm{d\Omega},$$

(18) $$\int_{\Omega }q\nabla \cdot w^{n}\;\mathrm{d\Omega}+L\left(v,q,p^{n},w^{n}\right)=0$$

for all $\left(\;v,q\right)\in {\left[X_{h}\right]}^{d}\times X_{h}$, with ${\left.\;v\;\right|}_{\Gamma }=\;0\;$, where L is a generic stabilization term to allow equal‐order finite element spaces $X_{h}$. Notice that the boundary terms coming from integration by parts vanish because ${\left.\;v\;\right|}_{\Gamma }=\;0\;$. Of course, other forms of the viscous term can also be used, such as

(19) $$\int_{\Omega }2\mu \nabla^{s}\;u^{n}:\nabla^{s}\;v\;\mathrm{d\Omega},$$

as proposed by Pacheco and Steinbach,^35^ or even the rotational form:

(20) $$\mu \int_{\Omega }\left(\nabla \times u^{n}\right)\cdot \left(\nabla \times v\right)\mathrm{d\Omega},$$

in which case we will use the notation STE${}_{\mathrm{int},\omega }$. Although there is no obvious a priori reason to pick one over the others – especially when u has been treated with solenoidal filters/projections^7^, ^21^, ^33^–, the “elasticity form” (19) has the advantage of allowing variable viscosity. Nevertheless, only the Laplacian (17) and rotational (20) forms will be considered herein.

### 3.3 Work‐energy balance estimators

When the goal is not to reconstruct the entire relative pressure field, but only to estimate an average pressure drop between two predefined planes, the effort in solving a boundary value problem for the pressure can actually be spared. Bertoglio et al.^18^ recently proposed using a virtual divergence‐free vector w as weighting function for the momentum Equation (1):

(21) $$\int_{\Omega }\;w\cdot \nabla p\;\mathrm{d\Omega}=\int_{\Omega }\;w\cdot \mu \nabla^{2}\;u\;\mathrm{d\Omega}-\int_{\Omega }\;w\cdot \rho \frac{\partial \;u\;}{\partial t}\;\mathrm{d\Omega}-\int_{\Omega }\;w\cdot \left[\left(\rho \nabla u\;\right)\;u\;\right]\mathrm{d\Omega}.$$

By taking $\;w\in H^{1}\left(\Omega \right)$ such that ∇⋅w=0 and ${\left.\;w\;\right|}_{\Gamma_{w}}=\;0\;$, integration by parts leads to:

(22) $$\int_{\Gamma_{i}\cup \Gamma_{o}}p\;w\cdot n\;\mathrm{d\Gamma}=\int_{\Gamma_{i}\cup \Gamma_{o}}\;w\cdot \left[\left(\mu \nabla u-\rho \;u⊗u\;\right)\;n\;\right]\mathrm{d\Gamma}+\int_{\Omega }\nabla w:\left(\rho \;u⊗u-\mu \nabla u\;\right)\mathrm{d\Omega}-\rho \int_{\Omega }\;w\cdot \frac{\partial \;u\;}{\partial t}\;\mathrm{d\Omega}.$$

Furthermore,

(23) $$\int_{\Gamma_{i}\cup \Gamma_{o}}\;w\cdot n\;\mathrm{d\Gamma}=\int_{\Gamma }\;w\cdot n\;\mathrm{d\Gamma}=\int_{\Omega }\nabla \cdot w\;\mathrm{d\Omega}=0.$$

Therefore, defining the virtual flow rate

(24) $$Q_{w}≔\int_{\Gamma_{i}}\;w\cdot n\;\mathrm{d\Gamma}=-\int_{\Gamma_{o}}\;w\cdot n\;\mathrm{d\Gamma}$$

and the w‐weighted pressure drop

(25) $$\delta_{w}p≔\frac{\int_{\Gamma_{i}}p\;w\cdot n\;\mathrm{d\Gamma}}{\int_{\Gamma_{i}}\;w\cdot n\;\mathrm{d\Gamma}}-\frac{\int_{\Gamma_{o}}p\;w\cdot n\;\mathrm{d\Gamma}}{\int_{\Gamma_{o}}\;w\cdot n\;\mathrm{d\Gamma}}\;$$

yields the integral momentum relative pressure (IMRP) estimator:

(26) $$\delta_{w}p^{n}=\frac{1}{Q_{w}}\left\{\;\int_{\Gamma_{i}\cup \Gamma_{o}}\;w\cdot \left[\left(\mu \nabla u^{n}-\rho \;u^{n}⊗u^{n}\right)\;n\;\right]\mathrm{d\Gamma}+\int_{\Omega }\nabla w:\left(\rho \;u^{n}⊗u^{n}-\mu \nabla u^{n}\right)\mathrm{d\Omega}-\frac{\rho }{\Delta t}\int_{\Omega }\;w\cdot \left(\;u^{n}-u^{n-1}\right)\mathrm{d\Omega}\right\},$$

with the virtual field typically pre‐computed as a Stokes flow. A variant of this method, the virtual work‐energy relative pressure (vWERP) estimator does not rely on integration by parts of the convective term:

(27) $$\delta_{w}p^{n}=\frac{1}{Q_{w}}\left\{\;\int_{\Gamma_{i}\cup \Gamma_{o}}\;w\cdot \left[\left(\mu \nabla u^{n}\right)\;n\;\right]\mathrm{d\Gamma}-\int_{\Omega }\mu \nabla w:\nabla u^{n}\;\mathrm{d\Omega}-\int_{\Omega }\;w\cdot \left[\frac{\rho }{\Delta t}\left(\;u^{n}-u^{n-1}\right)+\left(\rho \nabla u^{n}\right)\;u^{n}\right]\mathrm{d\Omega}\right\}.$$

In particular, setting w=u recovers the original work‐energy (WERP) estimator by Donati et al.^43^ Their method is less accurate than its virtual relatives, not only because of the squaring of the velocity data, but also due to limiting assumptions (e.g., non‐bifurcating geometry, immovable vessel walls with purely tangential velocity); it can also be inaccurate for low flow rates and has a provenly higher bias than other estimators (refer to Bertoglio et al.^18^ for a detailed discussion).

As in the STE, in principle any equivalent form of the viscous term can be used here. However, for the virtual work‐energy estimators, the rotational form brings a potential advantage. In practical applications of the vWERP estimator, the viscous boundary integral in Equation (27) (often denoted as $S_{e}$) is usually neglected, in order to mitigate errors related to inaccurate near‐wall measurements.^6^, ^8^, ^21^, ^32^ Although this is in practice a reasonable assumption, especially in typical cardiovascular flow regimes, it will be shown next how it is possible to construct a scheme where the boundary integral *naturally* vanishes. When applied to the virtual work‐energy equation, the rotational form of the stress divergence yields:

(28) $$-\mu \int_{\Omega }\;w\cdot \left[\nabla \times \left(\nabla \times u\;\right)\right]\mathrm{d\Omega}=-\mu \int_{\Omega }\left(\nabla \times w\;\right)\cdot \left(\nabla \times u\;\right)\mathrm{d\Omega}+\mu \int_{\Gamma }\left(\;n\times w\;\right)\cdot \left(\nabla \times u\;\right)\mathrm{d\Gamma}.$$

Compare the boundary term in Equation (28) to the one in Equation (27). In the original vWERP estimator, that term is proportional to the full trace ${\left.\;w\;\right|}_{\Gamma }$, so that eliminating the boundary integral would require a ${\left.\;w\;\right|}_{\Gamma }=\;0\;$ condition—thereby also annihilating the pressure term on the left‐hand side of (27). Conversely, the rotational version (28) contains not the full but only the tangential trace ${\left.\;n\times w\;\right|}_{\Gamma }$, so that the boundary integral can be easily eliminated by selecting a virtual field w with normal inflow and outflow conditions. For example, w can be chosen as the solution of the Stokes problem to find $\left(\;w,\lambda \right)$ satisfying

(29) $$\begin{array}{l} -\nabla^{2}\;w+\nabla \lambda =\;0\;\;\text{in}\;\Omega ,\\ \nabla \cdot w=0\;\text{in}\;\Omega ,\\ \;w=\varphi \;n\;\;\mathrm{on}\;\Gamma , \end{array}$$

with φ being a given scalar function on Γ with zero value on $\Gamma_{w}$ and zero mean on Γ (to guarantee global mass conservation for w). Then, it is simple to verify that the boundary integral in Equation (28) vanishes, since

(30) $${\left.\;n\times w\;\right|}_{\Gamma }=\varphi \;n\times n=\;0\;\text{for}\;\mathrm{any}\;\varphi .$$

In that case, one gets the rotational (vWERP${}_{\omega }$) estimator:

(31) $$\begin{array}{l} \delta_{\varphi }p^{n}=-\frac{1}{Q_{\varphi }}\left\{\mu \int_{\Omega }\left(\nabla \times w\;\right)\cdot \left(\nabla \times u^{n}\right)\mathrm{d\Omega}+\int_{\Omega }\;w\cdot \left[\frac{\rho }{\Delta t}\left(\;u^{n}-\;u^{n-1}\right)+\left(\rho \nabla u^{n}\right)\;u^{n}\right]\mathrm{d\Omega}\right\}, \end{array}$$

in which

$$\delta_{\varphi }p≔\frac{\int_{\Gamma_{i}}\mathrm{\varphi p}\;\mathrm{d\Gamma}}{\int_{\Gamma_{i}}\varphi \;\mathrm{d\Gamma}}-\frac{\int_{\Gamma_{o}}\mathrm{\varphi p}\;\mathrm{d\Gamma}}{\int_{\Gamma_{o}}\varphi \;\mathrm{d\Gamma}}\;\;\;Q_{\varphi }≔\int_{\Gamma_{i}}\varphi \;\mathrm{d\Gamma}=-\int_{\Gamma_{o}}\varphi \;\mathrm{d\Gamma}.$$

In particular, uniform inflow and outflow profiles can be used:

$$\varphi =\left\{\begin{array}{l} \begin{array}{cc} 0 & \mathrm{on}\;\Gamma_{w}, \end{array}\\ \begin{array}{cc} ‐1 & \mathrm{on}\;\Gamma_{i}, \end{array}\\ \begin{array}{cc} \frac{|\Gamma_{i}|}{|\Gamma_{o}|} & \mathrm{on}\;\Gamma_{o}. \end{array} \end{array}\right.$$

This specific choice of φ leads to:

$$\delta_{\varphi }p=\frac{1}{-|\Gamma_{i}|}\left(-\int_{\Gamma_{i}}p\;\mathrm{d\Gamma}+\frac{|\Gamma_{i}|}{|\Gamma_{o}|}\int_{\Gamma_{o}}p\;\mathrm{d\Gamma}\right)=\frac{1}{|\Gamma_{i}|}\int_{\Gamma_{i}}p\;\mathrm{d\Gamma}-\frac{1}{|\Gamma_{o}|}\int_{\Gamma_{o}}p\;\mathrm{d\Gamma}=\mathrm{\delta p},$$

that is, the weighted pressure drop is replaced by the standard mean pressure drop.

## 4 COMPUTATIONAL TESTS

In this section, various computational tests are carried out to evaluate the performance of the different estimators described so far. First, two‐dimensional problems with analytical solution are employed to assess the sensitivity of the different estimators with respect to noise. To isolate the influence of viscous and convective terms, the first and second examples are Navier–Stokes flows that also satisfy, respectively, the Stokes and Euler equations. Finally, 4D flow MRI data from an in‐vitro experiment will be used as a more practical test case. To assess noise sensitivity, the linearly interpolated velocity field is perturbed at each time step by an additive noise field with a normal distribution $N\left(0,\sigma^{2}\right)$, so as to simulate real measurements.^18^, ^20^ The noise level σ is relative to the (spatiotemporal) peak velocity. Given the large number of possible versions for the estimators depending on how viscous and convective terms are treated, the notation used throughout this article is given in Table 1.

**TABLE 1:** Notation employed to describe the three classes of estimators (Poisson, Stokes and work‐energy) with respect to different treatments of viscous and convective effects

Abbreviations	Viscous terms	Convective terms
PPE	Neglected	Convective form
PPE${}_{S}$	Stress divergence	Convective form
PPE${}_{\omega }$	Rotational form (integrated)	Convective form
PPE${}_{\mathrm{div}}$	Stress divergence	Divergence form
STE	Laplacian form (integrated)	Convective form
STE${}_{\mathrm{int}}$	Laplacian form (integrated)	Divergence form (integrated)
STE${}_{\mathrm{int},\omega }$	Rotational form (integrated)	Divergence form (integrated)
IMRP	Laplacian form (integrated)	Divergence form (integrated)
vWERP	Laplacian form (integrated)	Convective form
vWERP${}_{\omega }$	Rotational form (integrated)	Convective form

In the computations, first‐order Lagrangian finite elements are employed for the discretization of all quantities of interest. To solve the Stokes system required for the virtual work‐energy and Stokes estimators, a stabilization method is required to allow equal‐order discretization. For the two‐dimensional problems, which are solved in triangular meshes, the MINI element is used.^18^, ^44^ Since these elements are not defined for non‐simplicial meshes, in the 4D MRI case the stabilization method by Dohrmann and Bochev^45^ is employed — both techniques are absolutely stable, parameter‐free and simple to implement. All computations were done in MATLAB (MathWorks, Natick, MA) using its standard direct solvers.

### 4.1 Two‐dimensional Womersley flow

Given their known analytical solutions, two‐dimensional Womersley flows are a useful benchmark for pressure estimation methods.^7^, ^10^The setup considers a pulsatile velocity $\;u={\left(u,0\right)}^{Τ}$ given by

(32) $$u\;\left(x,y,t\right)=\left[1-{\left(\frac{y}{H}\right)}^{2}\right]U_{0}-U_{1}ℜe\left\{{\mathrm{ie}}^{\mathrm{i\omega t}}\left[1-\frac{\cosh\left(\mathrm{\alpha y}/H\right)}{\cosh\;\alpha }\right]\right\}$$

in a straight channel $\Omega =\left(0,L\right)\times \left(-H,H\right)$, where $\alpha =\sqrt{\mathrm{i\omega \rho}H^{2}/\mu }$ and ℜe denotes the real part of a complex number. The exact pressure field is (up to a function of time only)

(33) $$p\;\left(x,y,t\right)=-\left(\frac{2\mu U_{0}}{H^{2}}+\rho U_{1}\omega \;\cos\;\mathrm{\omega t}\right)x,$$

so that the analytical expression for the pressure drop is

(34) $$\mathrm{\delta p}\;\left(t\right)=\left(\frac{2\mu U_{0}}{H^{2}}+\rho U_{1}\omega \;\cos\;\mathrm{\omega t}\right)L.$$

**FIGURE 1:**
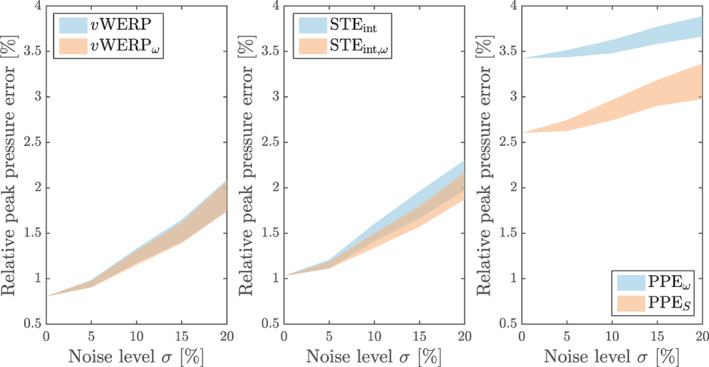
Womersley flow benchmark: noise sensitivity (95.45% confidence interval from 30 realizations) considering different versions of the Poisson, Stokes and virtual work‐energy relative pressure estimators

**FIGURE 2:**
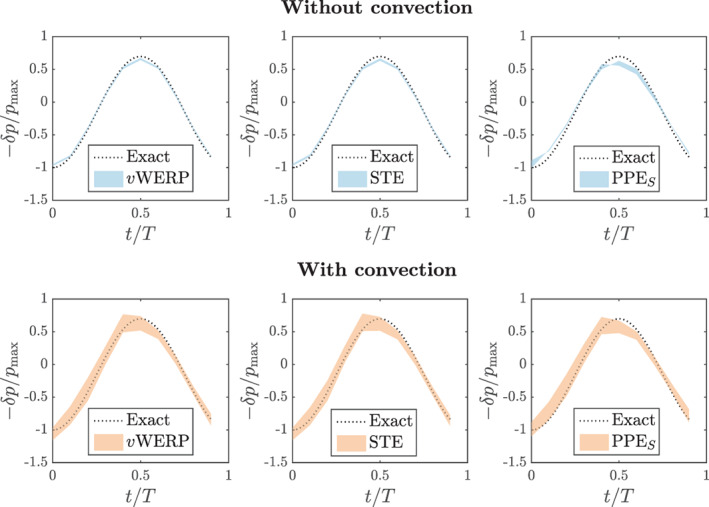
Womersley flow benchmark: relative pressure drop (20% noise level, 95.45% confidence interval) when including (lower row) or omitting (upper row) the convective term. The wider bands obtained when considering convection reveal a higher noise sensitivity stemming from convective terms than from viscous terms

The parameters used here are $U_{0}=U_{1}=0.25$ m/s, ω=2π rad/s, ρ=1000 kg/m^3^, μ=3.5 mPa s, and L=5H=25 mm. Since the Womersley flow is not only a Navier–Stokes but also a Stokes flow, the idea with this test case is to at first drop the convective term in the estimators so as to compare only the different treatments of the viscous term. The triangular mesh has a uniform spatial resolution of H/5, and the temporal resolution is set as T/10, with T=1 s being the pulsation period. The estimators' sensitivity to noise is evaluated through the relative peak error:

(35) $$\varepsilon_{\text{peak}}=\left|1-\frac{\max\left\{\mathrm{\delta p}\right\}}{\max\left\{\delta p_{\text{exact}}\right\}}\right|,$$

with 30 samples (cycles) computed per noise level. Two versions of each family of methods are considered: PPE${}_{S}$ and PPE${}_{\omega }$, vWERP and $v{\text{WERP}}_{\omega }$, and also the STE${}_{\mathrm{int}}$ in standard (grad‐grad) and rotational (curl‐curl) forms. Figure 1 shows the 95.45% confidence interval (corresponding to two standard deviations) of the peak error for different noise levels 0≤σ≤0.20. The results reveal good performance for all methods, all with less than 4% error even at 20% noise level. Although the integrated^35^ and the projected‐stress Poisson estimators display a similar variability, the error curve of the PPE${}_{S}$ is somewhat shifted downwards. This is the case even for σ=0, that is, the reason for the better performance of the PPE${}_{S}$ stems from its smaller discretization error. One possible explanation might be given in a very recent study by Araya et al.^46^: their theoretical estimates describe a suboptimal asymptotic convergence rate for the PPE${}_{\omega }$—although it is still unclear whether their analysis is sharp. For the other estimators, there are no significant changes whether using the Laplacian or rotational description of the viscous terms. The main reason for the small error levels in this example is the fact that the convective term has been “switched off”. When convection is included, the estimated pressure becomes visibly more sensitive to noise, as shown in Figure 2. This indicates that the quadratic nature of the convective term may be more harmful to the estimators' accuracy than the higher‐order derivatives of the viscous term—even for a flow regime with pronounced viscous effects such as this one. This difference will be even more expressive when considering the divergence form of the convective term, as shown in the next test case.

### 4.2 Two‐dimensional radial flow

The next test case considers a radial flow that solves both the Navier–Stokes and the Euler equations. This solution describes a pulsatile flow accelerating through a narrowing channel. The velocity and mean pressure drop between inlet and outlet are:

$$\;u\;\left(x,y,t\right)=\left(\begin{array}{c} V\left(t\right)x/\left(x^{2}+y^{2}\right)\\ V\left(t\right)y/\left(x^{2}+y^{2}\right) \end{array}\right),\;\mathrm{\delta p}\;\left(t\right)=\frac{\rho }{2}\left[\left(1-\frac{R^{2}}{r^{2}}\right)V^{2}\left(t\right)+R\dot{V}\left(t\right)\log\left(\frac{R^{2}}{r^{2}}\right)\right],$$

where R and r are the inlet and outlet radii, respectively, and $V\left(t\right)$ is a given pulsatility, which is chosen here as:

$$V\left(t\right)=-\left(1+A\;\sin\;\mathrm{\omega t}\right)U.$$

**FIGURE 3:**
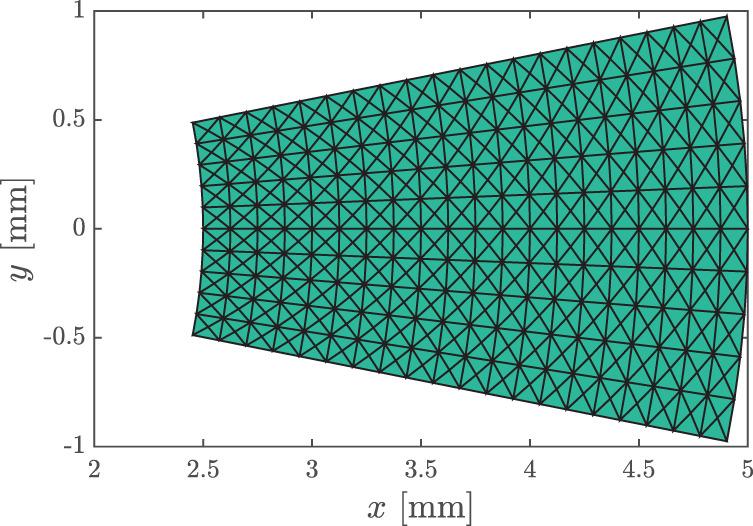
Radial flow benchmark: geometry and discretization used for the noise sensitivity study (flow direction: right to left)

**FIGURE 4:**
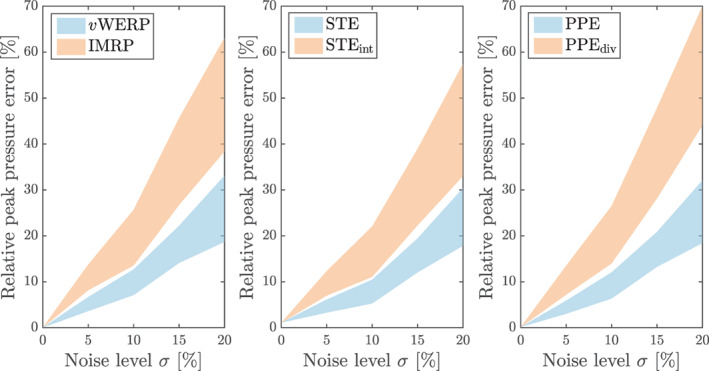
Radial flow benchmark: noise sensitivity (95.45% confidence interval from 30 realizations) considering different versions of the Poisson, Stokes and virtual work‐energy relative pressure estimators. The conservation form of the convective effects (IMRP, STE${}_{\mathrm{int}}$, PPE${}_{\mathrm{div}}$) is more sensitive to noise when compared to the standard (convective) form, for all three classes of estimators

The parameters set for this example are U=0.5 m/s, ω=2π rad/s, ρ=1000 kg/m${}^{3}$, R=2r=5 mm, and A=0.5, with an arc of π/8 rad. The discretized geometry is shown in Figure 3 (the flow happens from right to left), and the temporal resolution is again Δt=T/10=0.1 s. Now, the viscous term is dropped in the estimators so as to compare them solely with respect to the treatment of the convective term. The variants considered are the vWERP, IMRP, STE, STE${}_{\mathrm{int}}$, PPE and PPE${}_{\mathrm{div}}$ (cf. Table 1). The differences are now more expressive than in the previous case study: each of the methods is visibly more sensitive to noise when the divergence form of convection is used. Also notice that the errors here are at least one order of magnitude higher than in the previous example, once more indicating the higher noise sensitivity caused by convective (in comparison to viscous) terms. It is important to remark that, in practice, 4D flow data are acquired spatially in a Cartesian‐like manner, so that boundaries will in general not be resolved as in Figure 3; a more realistic setup will be considered in the next test case (Figure 4).

### 4.3 In‐vitro flow in giant intracranial aneurysm phantom

The next test case considers a more realistic scenario using real 4D flow MRI measurements. For an in‐depth study on the flow mechanism in intracranial aneurysms, Amili et al.^30^ constructed a 3D‐printed replica of a giant, pre‐treatment aneurysm^14^ in an internal carotid artery (ICA). The geometry was scaled (enlarged) by a factor of 2.0 to enable more accurate measurements, and the remaining parameters were adjusted to have an in‐vitro experiment matching the physiological Reynolds and Womersley numbers. The experiment had a velocity encoding of 0.8 m/s along all directions, a $15^{\circ }$ flip angle and a spatial resolution of 0.6×0.6×0.6 mm${}^{3}$. The pulsation period was T=7.92 s, with 16 acquisitions per cycle (Δt=0.495 s). The fluid properties were measured as ρ=1060 kg/m${}^{3}$ and μ=2.12 mPa s, and further details on the experimental setup can be found in the original article.^30^

**FIGURE 5:**
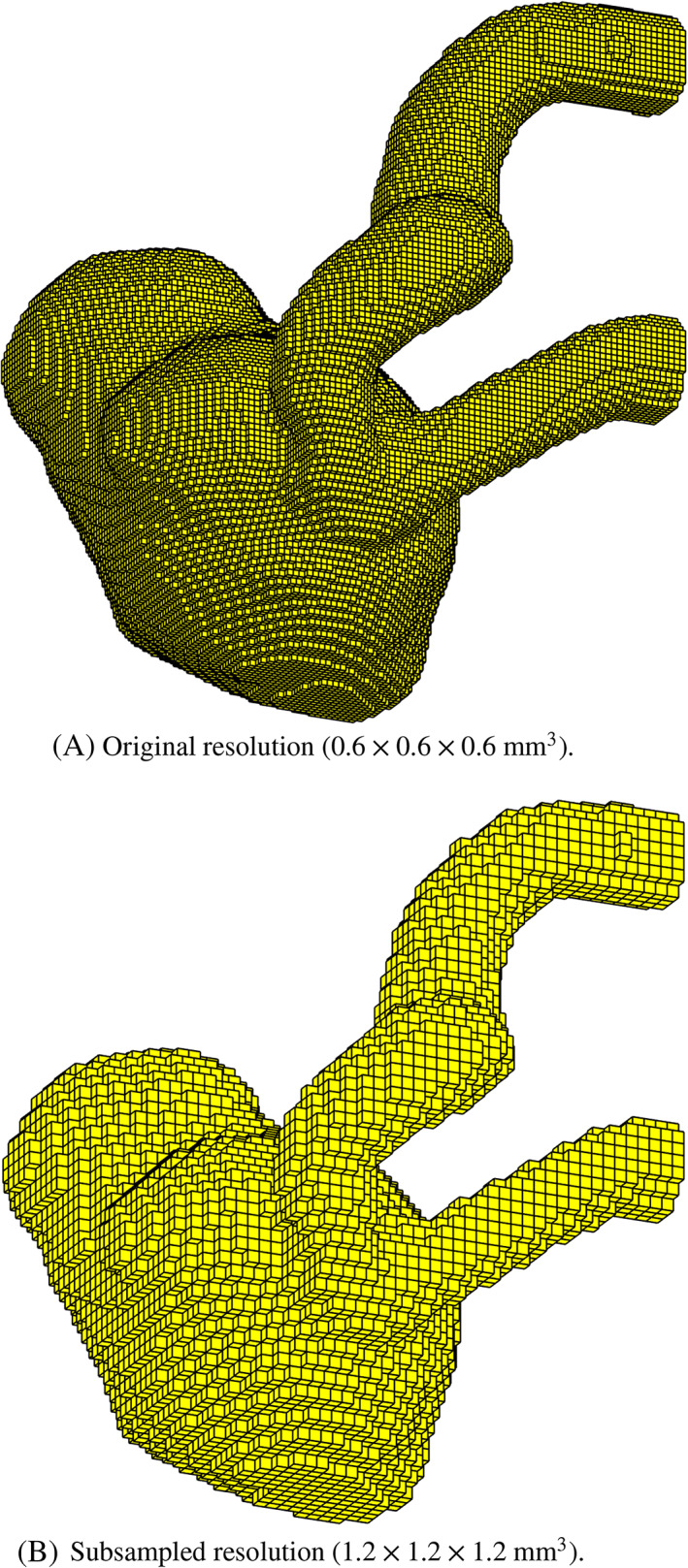
Hexahedral meshes constructed from the in‐vitro data by Amili et al.^30^

**FIGURE 6:**
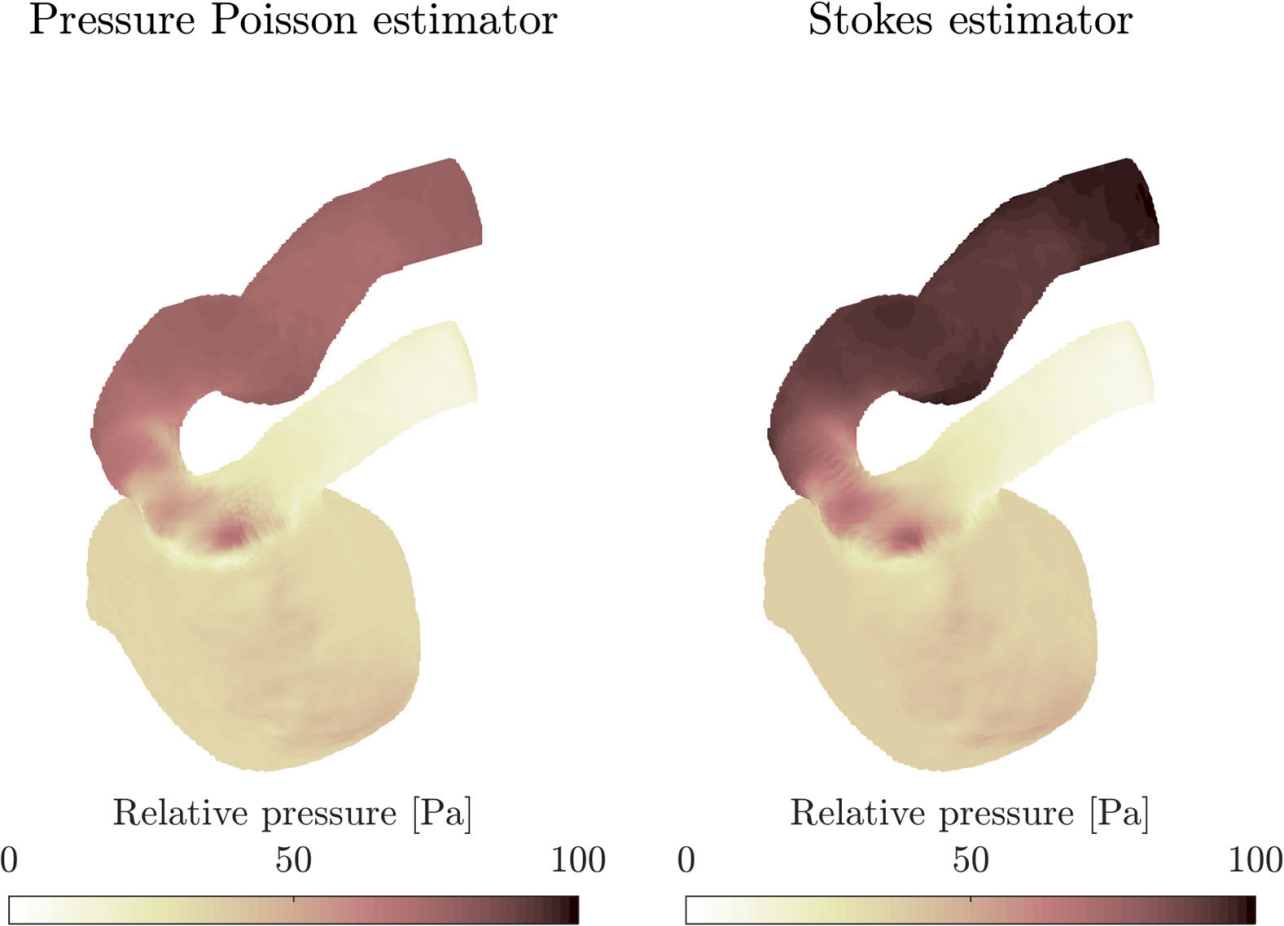
Relative systolic pressure distribution computed from 4D flow measurements in an in‐vitro ICA aneurysm^30^ using the PPE_*S*_ and STE. As often observed, the Poisson estimator computes lower relative pressure magnitudes

The first step for the pressure computation is to extract a finite element mesh from the data. For this, a tensor‐product hexahedral grid is first constructed directly from the voxelated data. Then, the elements corresponding to voxels outside the aneurysm are deleted (the published data^30^ contain a mask assigning a “number/not‐a‐number” flag to each data point). Finally, the mesh is truncated at x=13 cm to eliminate the artificial prolongations of the artery used in the experiment (cf. Figure 1 therein^30^). No erosion^30^ or deletion^9^ of wall voxels was performed. The final hexahedral mesh, depicted in Figure 5A, contains approximately 2 × 10^5^ elements and nodes. A subsampled version with half of the original spatial resolution is also considered for the last test, see Figure 5B. The relative pressure fields computed at systole by the pressure Poisson and Stokes estimators are illustrated in Figure 6, which shows—as usual—that the PPE estimates a lower relative pressure.

**FIGURE 7:**
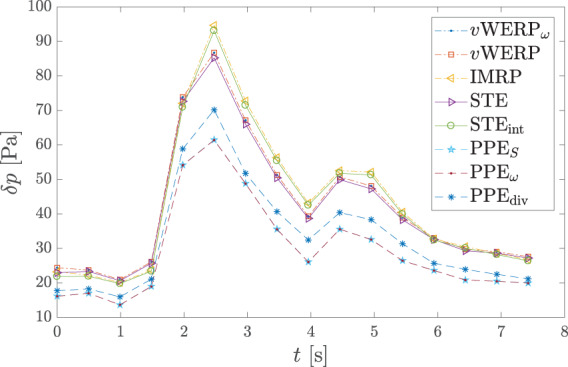
Pressure drop computed from 4D flow measurements in an in‐vitro ICA aneurysm.^30^ The Stokes and vWERP estimators agree very well with each other, and so do the IMRP and STE${}_{\mathrm{int}}$ (since both use the same integrated form of the convective term). There is virtually no difference between the vWERP and vWERP${}_{\omega }$ estimates, which confirms that the surface integral $S_{e}$ is negligible for this flow. Also between PPE${}_{S}$ and PPE${}_{\omega }$ there is hardly any difference, whereas using the divergence form of convection (PPE${}_{\mathrm{div}}$) increases the pressure drop, as in the other two families of estimators

Figure 7 depicts the mean pressure drop $\mathrm{\delta p}\;\left(t\right)$ between the inlet and outlet planes. The Stokes and virtual work‐energy estimators show excellent mutual agreement, with less than 2% difference in the peak systolic value. The same goes for the IMRP and STE${}_{\mathrm{int}}$, both of which use the same integrated version of the convective term. Although there are no reference measurements here, Bertoglio et al.^18^ also noted that the (integrated) divergence form of convection increases the estimated pressure for the STE. The results in Figure 7 confirm that this is also the case for the other two families of estimators. The vWERP and vWERP${}_{\omega }$ estimates are virtually indistinguishable, which confirms that, as often assumed in the literature,^6^, ^8^, ^21^, ^32^ the viscous boundary term $S_{e}$ is negligible also for this test case. There is also hardly any difference between the projected‐stress and integrated Poisson estimators, which is most likely due to the rather high resolution of the data (the PPE${}_{\omega }$ typically has a higher truncation error, see Figure 1 [right] and also Ref. 46).

**FIGURE 8:**
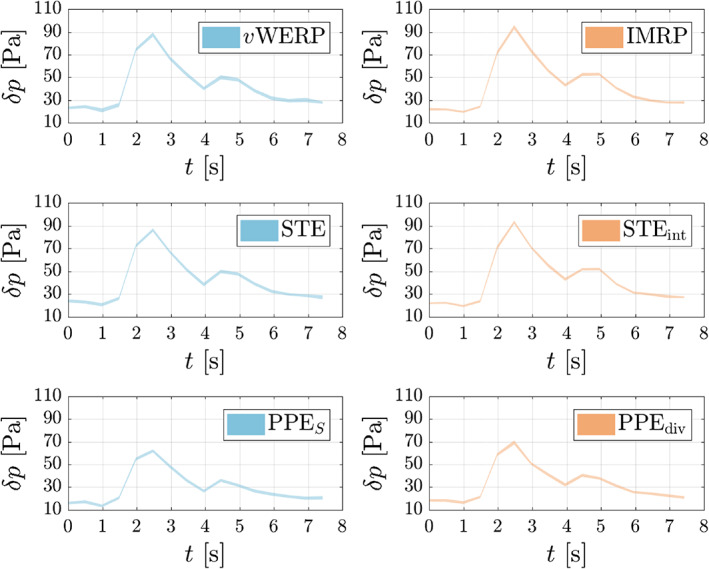
Pressure drop (95.45% confidence interval from 30 realizations) computed from 4D flow measurements in an in‐vitro ICA aneurysm,^30^ with artificially added noise (σ=0.20). The divergence variants of the convective term (right column) estimate a higher pressure drop in all cases, but there is no “clear winner” with respect to noise sensitivity

**FIGURE 9:**
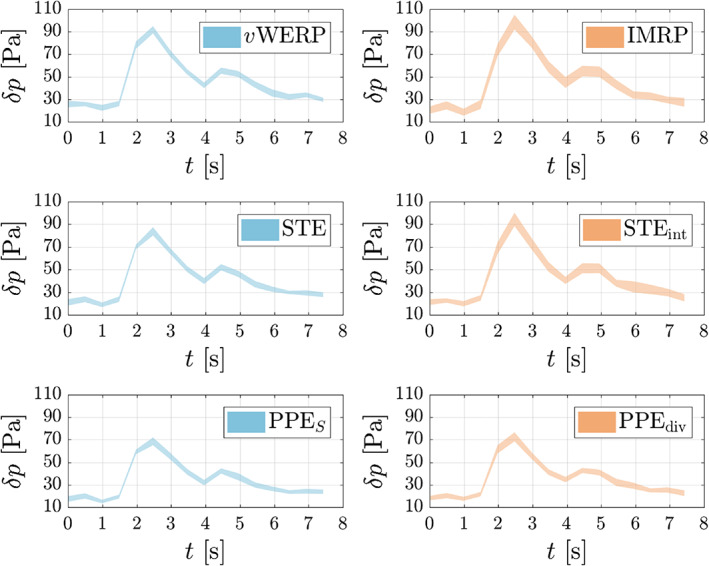
Pressure drop (95.45% confidence interval from 30 realizations) computed from 4D flow measurements in an in‐vitro ICA aneurysm,^30^ with artificially added noise (σ=0.20) and spatial subsampling, cf. Figure 5B. The divergence variants of the convective term (right column) estimate a higher pressure drop in all cases, but are more sensitive to noise

Next, as done by Nolte et al.,^9^ noise is artificially added to the data in order to compare the estimators with respect to the different treatment of the convective term. The results, depicted in Figure 8, show rather small variability even for the high noise level applied (σ=0.20). This is due to the relatively high resolution of this dataset, which considers an enlarged aneurysm phantom (error bands for in‐vivo measurements are typically higher). For this reason, no clear winner in terms of noise sensitivity can be picked from the results in Figure 8, with all variants displaying a comparable variability. Then, to yield a more realistic comparison, a spatial subsampling of the original data is applied, with half of the original resolution (cf. Figure 5B). The results, depicted in Figure 9, are now more conclusive. At the pressure peak, the standard deviation of the STE${}_{\mathrm{int}}$ is 62% larger than that of the STE, and the difference is even larger (106%) between the IMRP and vWERP estimators. For the PPE, though, the divergence form of convection only led to a 16% increase in the standard deviation. These results confirm the trend observed in the in‐silico test case: the divergence form of the convective term is indeed more sensitive to noisy velocity data.

## 5 DISCUSSION

### 5.1 Main findings

This study compared three families of relative pressure estimators (Poisson, Stokes and virtual work‐energy balance) with different ways of treating viscous and convective terms. Classical treatments as well as two new ones were investigated. The in‐silico tests indicate that, for all three classes considered here, the integrated forms (PPE${}_{\omega }$, STE${}_{\mathrm{int}}$, and IMRP) yield larger errors for noisy data. Another key finding here is that the noise sensitivity of the convective term can be considerably higher than that of the viscous term (despite the latter's second‐order derivatives) even for cerebrovascular flow regimes with increased viscous contribution. Moreover, the investigation with in‐vitro 4D MRI data confirmed the well‐known underestimation bias of pressure Poisson estimators (in different variants). Notice, though, that new ideas and techniques have recently emerged to increase the PPE's accuracy. For instance, Araya et al.^46^ noted that increasing the polynomial order of the pressure interpolant can mitigate the underestimation of peak systolic pressures. A similar effect can be achieved by removing the outer layer of voxels, as recently reported by Nolte et al.^9^ A more accurate computation of the viscous term can also be achieved by reconstructing the velocity field near the walls.^3^

### 5.2 Treatment of viscous terms

The (herein introduced) projected‐stress pressure Poisson estimator (PPE${}_{S}$) improves the accuracy of the viscous pressure drop, although this improvement is likely to be overshadowed by convection‐related effects in clinical applications. However, the viscous component per se can be a potential biomarker for aortic diseases,^3^ in which case a more accurate computation of viscous effects becomes relevant. In particular, the near‐wall velocity reconstruction technique proposed by Donati et al.^3^ can be combined with the present viscous modifications to better capture viscous pressure drops. In such a context, the boundary‐integral‐free modification proposed herein for the virtual estimators may prove advantageous. Although whether it could have an impact in clinical practice is unclear at the moment, the vWERP${}_{\omega }$ estimator has no added cost or complexity in comparison to the classical versions, and has the advantage of not requiring prior phenomenological knowledge on the flow regime by the user, since the boundary integral vanishes exactly for any flow.

### 5.3 Treatment of convection terms

The convective effects in all estimators can be treated in either convective (standard) or divergence/conservation form. Using the latter in Stokes and work‐energy estimators enables integration by parts, thus eliminating derivatives. Different test cases reveal increased noise sensitivity when shifting from the convective to the conservation form—in all three families of methods considered. On the other hand, the study with in‐vitro data showed a larger pressure drop estimated by the conservation variants. Although there are no reference measurements here to judge such increase in estimated pressure, this behaviour is in line with the findings of Bertoglio et al.,^18^ who reported that the integrated variants can mitigate the pressure underestimation caused by low‐resolution data.

### 5.4 Limitations

The present work is application‐driven, yet still fundamental and, up to a certain extent, also theoretical. The test cases reveal insightful information and trends, but the absence of reference in‐vivo data limits the reach of some of the conclusions drawn here. Moreover, the improved computation of viscous terms does not solve the dependency of relative pressure field estimators (PPE, STE) on the defined geometrical domain.^8^, ^9^ In fact, viscous effects are most significant near vessel walls, which are often not (accurately) included in the final computational domain.^3^, ^9^ Therefore, the methods presented here could and should be combined with techniques aimed at improving near‐wall velocity accuracy.^3^

## 6 CONCLUDING REMARKS AND FUTURE DEVELOPMENTS

The purpose of this work has been twofold: from one side, to compare different treatments of viscous and convective effects in relative pressure estimation and, from another, to propose modifications in the viscous terms to circumvent surface integrals. The in‐silico and in‐vitro datasets considered here lie in the typical physiological regime of cerebrovascular flows, where viscous forces are more significant than in, for example, aortic applications. In the aorta, it is known that viscous terms are usually negligible if compared to inertial ones,^1^, ^18^ therefore noise‐related errors due to viscous second‐order derivatives are also typically small (see e.g., Figure 4 in Ref. 1). What is now shown here is that this applies also to flows with lower Reynolds and Womersley numbers. In fact, even in a Womersley flow with no (physical) convective contribution whatsoever, viscous effects can be the least significant source of error. Moreover, using the integrated forms of Poisson, Stokes or virtual work estimators tends to increase noise sensitivity, in spite of their reduced order of differentiation on the velocity data.

As for the modifications introduced here, the in‐silico tests showed that the projected‐stress PPE can improve the accuracy of the viscous pressure drop. It has also the advantage of allowing for local viscosity variations, which could improve the computation of turbulence‐related pressure drops stemming from eddy viscosities.^33^ The present rotational vWERP, on the other hand, did not provide tangible differences with respect to the standard variant. It can still be seen, however, as more robust in theory, by consistently eliminating (rather than neglecting) viscous surface integrals regardless of the flow regime—without making any assumptions on the relative magnitudes of different virtual work terms. Whether this will be advantageous for flows of clinical interest, however, is not known at the moment.

Two ramifications are planned for the continuation of this study. The first and obvious one is to assess the modified variants presented here in combination with in‐vivo data. The second one involves a more technical aspect. One of the reasons normally attributed to the underestimation bias of pressure Poisson estimators are their increased regularity (continuity) requirements on the pressure field.^9^ In this context, on‐going work includes the implementation of an ultra‐weak finite element method for the PPE.^47^ In such a framework, integration by parts can be applied to reduce the pressure regularity requirements down to the same ones as in the Stokes estimator, thereby potentially increasing the accuracy of the PPE in low‐resolution grids.

## Data Availability

Data sharing is not applicable to this article as no new data were created or analyzed in this study.
